# Embryonic Stem Cell Growth Factors Regulate eIF2α Phosphorylation

**DOI:** 10.1371/journal.pone.0139076

**Published:** 2015-09-25

**Authors:** Kyle Friend, Hunter A. Brooks, Nicholas E. Propson, James A. Thomson, Judith Kimble

**Affiliations:** 1 Department of Biochemistry, University of Wisconsin-Madison, Madison, Wisconsin, 53706, United States of America; 2 Department of Chemistry and Biochemistry, Washington and Lee University, Lexington, Virginia, 24450, United States of America; 3 The Morgridge Institute for Research, 309 North Orchard Street, Madison, Wisconsin, 53715, United States of America; 4 Department of Cell and Regenerative Biology, University of Wisconsin School of Medicine and Public Health, Madison, Wisconsin, 53706, United States of America; 5 Howard Hughes Medical Institute, University of Wisconsin-Madison, Madison, Wisconsin, 53706, United States of America; University of Kansas Medical Center, UNITED STATES

## Abstract

Growth factors and transcription factors are well known to regulate pluripotent stem cells, but less is known about translational control in stem cells. Here, we use embryonic stem cells (ESCs) to investigate a connection between ESC growth factors and eIF2α-mediated translational control (eIF2α phosphorylation promotes protein expression from mRNAs with upstream open-reading frames, or uORFs). We find abundant phosphorylated P-eIF2α (P-eIF2α) in both pluripotent mouse and human ESCs, but little P-eIF2α in ESCs triggered to differentiate. We show that the growth factors LIF (leukemia inhibitory factor) and BMP4 (bone morphogenic protein 4) both maintain P-eIF2α in mESCs, but use distinct mechanisms: LIF inhibits an eIF2α phosphatase whereas BMP4 activates an eIF2α kinase. The mRNAs encoding the pluripotency factors Nanog and c-Myc possess uORFs while Oct4 mRNA does not. We find that salubrinal, a chemical that increases eIF2α phosphorylation, promotes Nanog and c-Myc expression, but not Oct4 expression. These experiments connect ESC growth factors to eIF2α phosphorylation and suggest a chemical substitute for LIF to enhance Nanog and c-Myc expression.

## Introduction

Embryonic stem cells (ESCs) maintain a pluripotent state that is controlled by regulatory networks. Best established are the networks of epigenetic chromatin modifications and transcriptional regulation (reviewed in [1, 2]), but translational regulation can also influence ESC pluripotency. For example, mouse Lin28 downregulates *let-7* miRNA to maintain pluripotency [3], and human LIN28 can be added with the OCT4, SOX2 and NANOG transcription factors to induce pluripotency in human somatic cells [4]. Also, miRNAs can induce somatic cells to pluripotency, as with the miR302/367 cluster [5]. In addition, a general mechanism of translational regulation affects pluripotency: in mouse ESCs, active eIF4E cap-binding protein represses translation, but that repression is alleviated upon differentiation [6–8].

A different mechanism of translational control uses upstream open-reading frames (uORFs) to inhibit translation of the coding sequence (CDS) in the same mRNA [9]. Remarkably, mESCs initiate translation at uORFs in the Nanog and c-Myc mRNAs [10] despite the importance of these key pluripotency factors [11, 12]. This paradox suggests that ESCs likely regulate translation in mRNAs containing uORFs. The classic example of uORF regulation involves a yeast mRNA encoding the GCN4 transcription factor (reviewed in [9]). During translation initiation, ribosomes scan in the 5' to 3' direction to find start codons, preferentially initiating translation at the first start codon encountered, although a consensus sequence influences start codon usage [13, 14]. In the GCN4 mRNA, multiple uORFs are located upstream of the CDS; ribosomes initiate, elongate and terminate translation on the first uORF. Then ribosomes can reinitiate translation downstream on either the other uORFs or the CDS. This reinitiation event is controlled by the phosphorylation and dephosphorylation of the translation initiation factor, eIF2α. Phosphorylated eIF2α (P-eIF2α) slows the rate at which ribosomes reinitiate, allowing the ribosome to scan to the downstream CDS, promoting translation [15, 16]. More recent studies have confirmed and extended these findings in mammals, observing that P-eIF2α typically enhances CDS translation from mRNAs containing uORFs [17].

The enzymes regulating eIF2α phosphorylation include both kinases and phosphatases. The eIF2α kinases, four from the same family, act in response to cellular stresses (*e*.*g*. nutrient deprivation, hypoxia and viral infection) [18–20]. The eIF2α phosphatase complexes contain either GADD34 [21] or CReP (Constitutive Repressor of eIF2α Phosphorylation) [22] and oppose eIF2α phosphorylation. GADD34 is induced upon stress, whereas CReP is more ubiquitously expressed, although CReP expression can also be regulated [23]. Relevant to this work, CReP and GADD34 can be inhibited specifically with salubrinal, increasing P-eIF2α abundance [24]. The active eIF2α phosphatase complexes contain a catalytic subunit, protein phosphatase 1c (PP1), which is not inhibited by salubrinal [24]. However, salubrinal inhibits PP1 complexed with either CReP or GADD34, likely by disrupting the complex [24].

Here, we investigate a role for eIF2α phosphorylation in ESCs and probe the connection between growth factors and eIF2α. We report that both human and mouse ESCs contain abundant P-eIF2α and that P-eIF2α decreases dramatically upon differentiation. We then show that the growth factors LIF (leukemia inhibitory factor) and BMP4 (bone morphogenic protein 4) act synergistically to maintain P-eIF2α: LIF downregulates the CReP eIF2α phosphatase, and BMP4 activates the eIF2α kinase, Protein Kinase regulated by RNA (PKR). Finally, we show that chemical induction of P-eIF2α, using salubrinal, maintains expression of Nanog and c-Myc proteins in mouse ESCs cultured without LIF. Together, our results connect ESC growth factors to the control of eIF2α phosphorylation in ESCs and identify a chemical tool that promotes ESC maintenance.

## Materials and Methods

### Cell culture

Mouse embryonic stem cells (line E14Tg2a, MMRRC) were propagated in ESGRO complete medium supplemented with GSK3β inhibitor (Cat. No: SF001-500P, Millipore, Billerica, MA) at 37°C in 5% CO_2_ on gelatin-coated tissue culture plates. Medium was exchanged daily, and cells were passaged with Accutase (Millipore, Billerica, MA) every 3 days at a 1:8 split ratio.

For experiments involving removal of cytokines, ESGRO basal medium (Cat. No: SF002-500, Millipore, Billerica, MA) with GSK3β inhibitor was supplemented either with recombinant BMP4 (10 ng/mL, R&D Systems, Minneapolis, MN) or recombinant LIF (Sigma, St. Louis, MO, 10 ng/mL unless otherwise indicated). Note that in all cases, GSK3β inhibitor was included with ESCs. All protein and RNA samples were collected 24 h after medium exchange. Salubrinal (Millipore, Billerica, MA) was resuspended in DMSO at a stock concentration of 1 mM. Salubrinal was then added to ESGRO basal medium supplemented with GSK3β inhibitor and BMP4 at a final concentration of 10 nM and 100 nM. In all cases, DMSO was added in the same amount as a control. Samples were collected 24 h after medium exchange with the exception of the samples for prolonged salubrinal treatment: medium with 100 nM salubrinal was exchanged daily for four days with a split on day 3. There was no difference in doubling time or apoptosis observed in cells grown with salubrinal or in ESGRO complete medium.

The human ES cell line H1 was cultured in E8 medium as previously described [25]. Mesendoderm differentiation was also performed as previously described [26], but cells were harvested after one day of growth rather than after multiple days of culture.

### Antibodies

Anti-eIF2α, P-eIF2α (Ser51), eIF4G, P-eIF4G (Ser1108), eIF4E, P-eIF4E (Ser209) and Oct4 antibodies were obtained from Cell Signaling Technology (Danvers, MA). Anti-mNanog was obtained from R&D Systems (Minneapolis, MN). Anti-hNANOG and pPKR (Thr446) were obtained from Millipore (Billerica, MA), and anti-Actin (mouse C4) and c-Myc (polyclonal) were obtained from Sigma (St. Louis, MO). Anti-PKR and Nck1/2 were obtained from Santa Cruz Biotechnology (Dallas, TX). Anti-CReP was obtained from ProteinTech (Chicago, IL).

### Western blots

**Fig 1:**
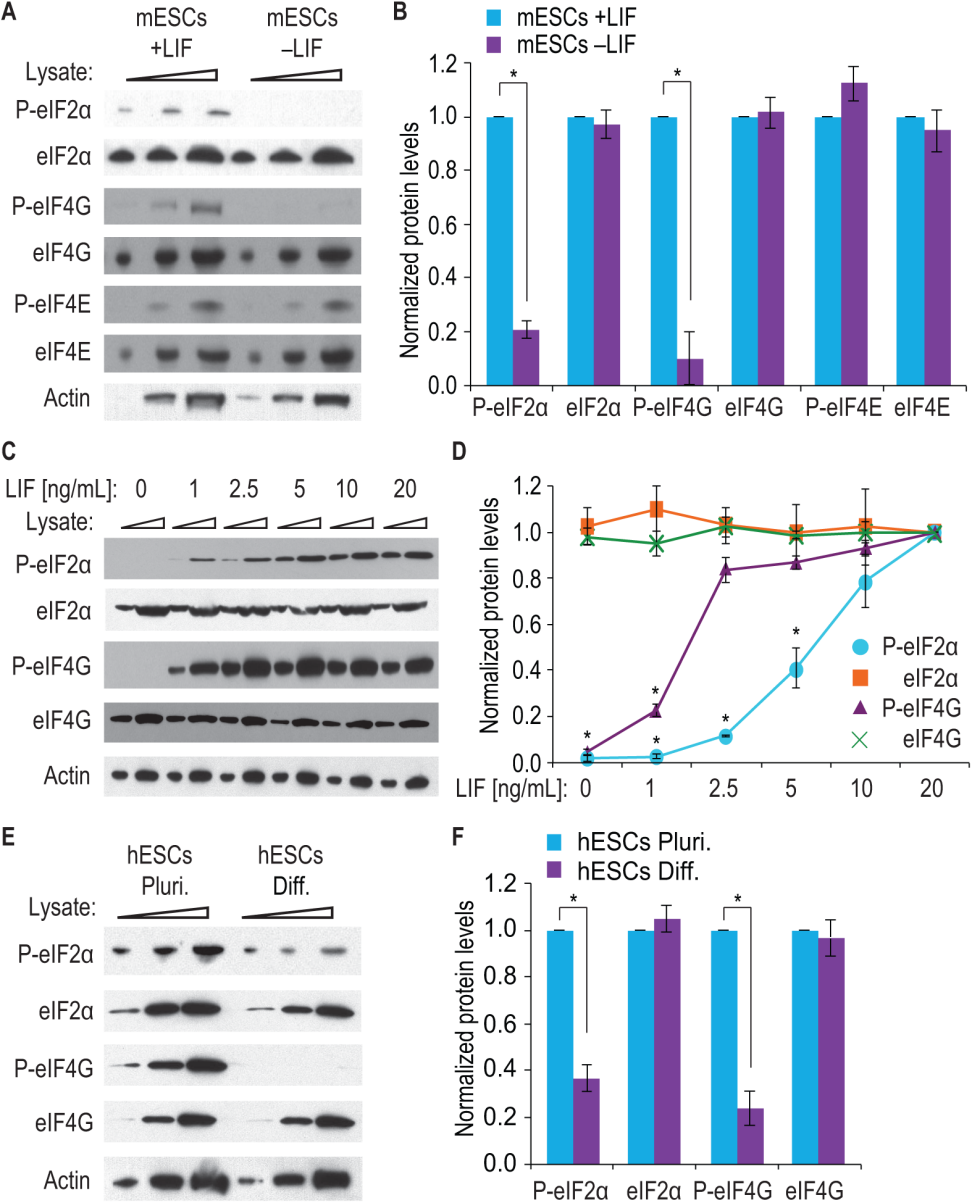
Phosphorylated eIF2α and eIF4G in mESCs and hESCs. (A) mESCs were cultured either with LIF (mESCs +LIF) or without LIF for 24 h (mESCs–LIF). Increasing amounts of cell lysate (indicated by triangles) were separated by 4–12% gradient SDS-PAGE and indicated proteins visualized by western blot. P-eIF2α, P-eIF4G and P-eIF4E, phosphorylated eukaryotic translation initiation factors; eIF2α, eIF4G and eIF4E, total eukaryotic translation initiation factors. Actin was probed as a loading control. (B) Three biological replicates, prepared as in (A), were quantitated. Data were normalized to values from mESCs cultured with LIF (mESCs +LIF). Both P-eIF2α and P-eIF4G are downregulated in mESCs cultured without LIF, whereas P-eIF4E is unchanged. Error bars represent s.d. (*, p < 0.01 compared to mESCs +LIF samples). (C) mESCs were cultured for 24 h with various concentrations of LIF (10 ng/mL is standard); increasing amounts of cell lysate (indicated by triangles) were separated by SDS-PAGE and analyzed by western blot for indicated proteins. (D) Three biological replicates, prepared as in (C), were quantitated. Data were normalized to values from mESCs cultured with 20 ng/mL LIF. Increasing LIF promotes increasing phosphorylation of eIF2α and eIF4G. Note that LIF titration promotes eIF4G phosphorylation prior to eIF2α phosphorylation. Error bars represent s.d. (*, p < 0.01 compared to 10 ng/mL LIF samples). (E) hESCs were cultured for 24 h with FGF2 and TGF-β (hESCs Pluri.) or under conditions that induce mesendoderm differentiation (hESCs Diff.). Increasing amounts of cellular lysate (indicated by triangles) were separated by SDS-PAGE and western blotted for indicated proteins. (F) Three biological replicates, prepared as in (E), were quantitated. Data were normalized to values from hESCs cultured with FGF2 and TGF-β (hESCs Pluri.). eIF2α and eIF4G are phosphorylated in pluripotent hESCs more than in early differentiating hESCs (hESCs Diff.). Error bars represent s.d. (*, p < 0.01 compared to hESCs Pluri. samples).

**Fig 2:**
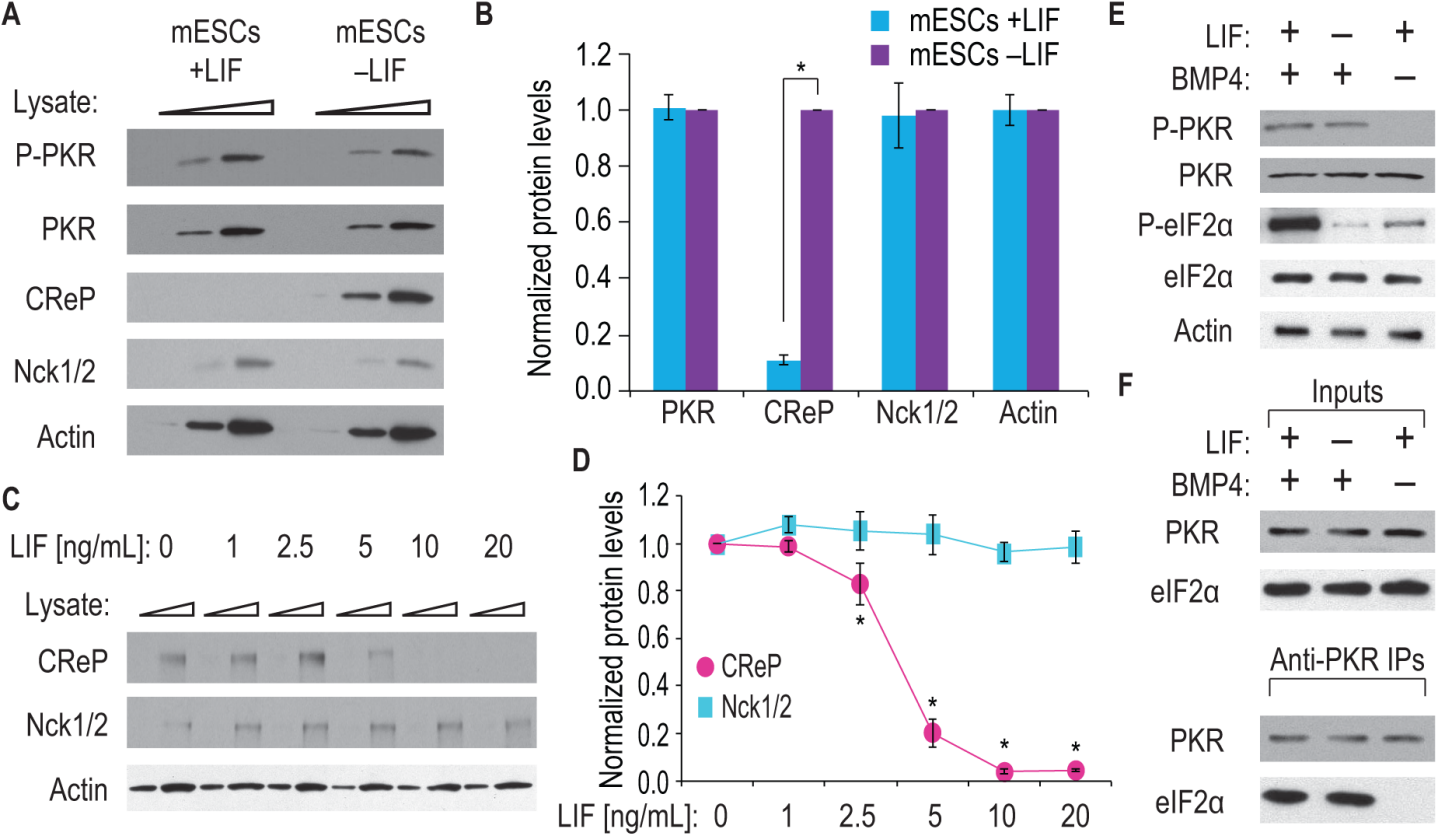
LIF and BMP4 enhance eIF2α phosphorylation. (A) mESCs were cultured for 24 h either with LIF (mESCs +LIF) or without LIF (mESCs–LIF). Increasing amounts of cell lysate (indicated by triangles) were separated by SDS-PAGE and proteins visualized by western blot. PKR was uniformly present in both samples, with no difference in active PKR levels (P-PKR). CReP levels were low in mESCs cultured with LIF and upregulated in mESCs cultured without LIF. Nck1/2 levels were uniform. Actin is a loading control. (B) Three biological replicates were prepared as in (A) and quantitated. Data were then normalized to values from mESCs grown without LIF. CReP levels increased when LIF was withdrawn, whereas PKR and Nck1/2 levels were unchanged. Error bars represent s.d. (*, p < 0.01 compared to mESCs–LIF samples). (C) mESCs were cultured for 24 h with increasing concentrations of LIF (10 ng/mL is standard). Increasing amounts of cell lysate (indicated by triangles) were separated by SDS-PAGE and western blotted for CReP, Nck1/2 and Actin. LIF downregulates CReP, but not Nck1/2 or Actin. (D) Three biological replicates were prepared as in (C) and quantitated. Data were normalized to values from mESCs cultured without LIF. Increasing LIF decreases CReP levels, but does not affect Nck1/2. Error bars represent s.d. (*, p < 0.01 compared to 0 ng/mL LIF samples). (E) mESCs were cultured for 24 h with or without LIF and with or without BMP4 (indicated by + and–signs). Higher levels of active P-PKR were seen with BMP4, less with BMP4 removed. P-eIF2α levels were highest in mESCs grown with both LIF and BMP4. Total PKR and eIF2α levels were constant as was Actin, probed as a loading control. (F) The presence of active P-PKR was assayed by co-IP for PKR and its substrate eIF2α. mESCs were cultured as in (E); Western blots visualized PKR and eIF2α in total cell lysates (Input; upper panels) or PKR IPs (Anti-PKR IP; lower panels). eIF2α is present in all inputs (upper panel), but eIF2α co-IPs with PKR only in cells treated with BMP4 (lower panel).

**Fig 3:**
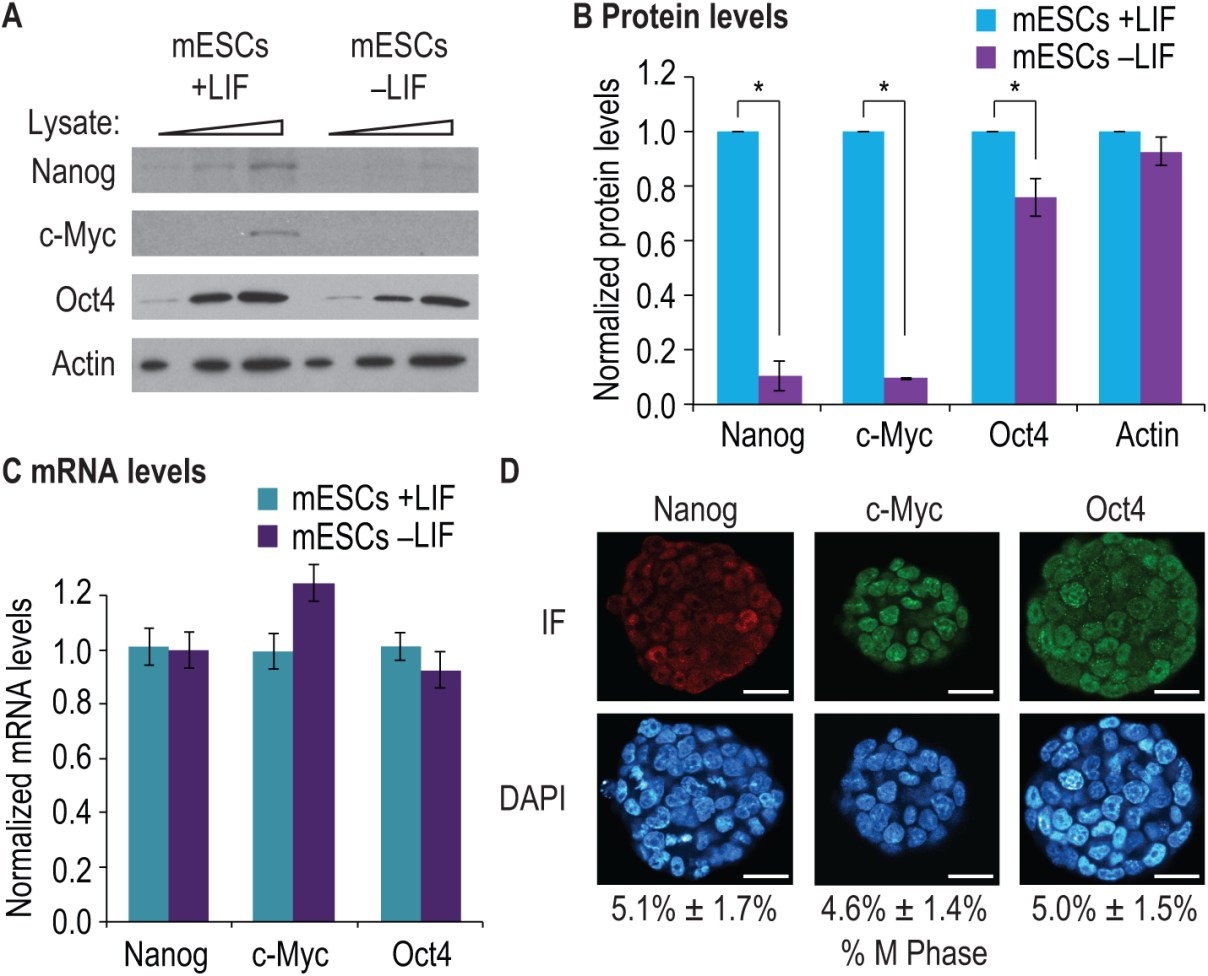
eIF2α phosphorylation correlates with Nanog and c-Myc expression. (A) mESCs were cultured for 24 h either with LIF (mESCs +LIF) or without LIF (mESCs–LIF). Increasing amounts of cell lysate (indicated by triangles) were separated by SDS-PAGE and western blotted for indicated proteins. Nanog and c-Myc protein levels decreased after LIF withdrawal, whereas Oct4 levels were more modestly decreased when LIF was withdrawn. Actin served as a loading control. (B) Three biological replicates were prepared as in (A) and quantitated. Data were then normalized to values from mESCs grown with LIF. Error bars represent s.d. (*, p < 0.01 compared to mESCs +LIF samples). (C) RNA was prepared from mouse ESCs cultured as in (A) and RT-qPCR used to assay abundance of indicated RNAs. Loading was normalized to a control probe set (eEF1A mRNA). To provide relative mRNA abundance, data were normalized to mESCs grown with LIF. Nanog, c-Myc and Oct4 mRNA levels were unaltered in mESCs grown with or without LIF. Error bars represent s.d. (D) Mouse ESCs were cultured with LIF and BMP4. Colonies were then fixed and stained for the indicated proteins (IF samples); DAPI staining was used to visualize nuclear DNA. Nanog, c-Myc and Oct4 proteins were detectable in all cells within a colony. Mouse ESC cell divisions were confirmed by counting and calculating the percentage of cells in M phase for each sample, as shown below each representative colony. Scale bars: 50 μm. Three separate mESC cultures were used to quantitate M phase cells (s.d. shown).

**Fig 4:**
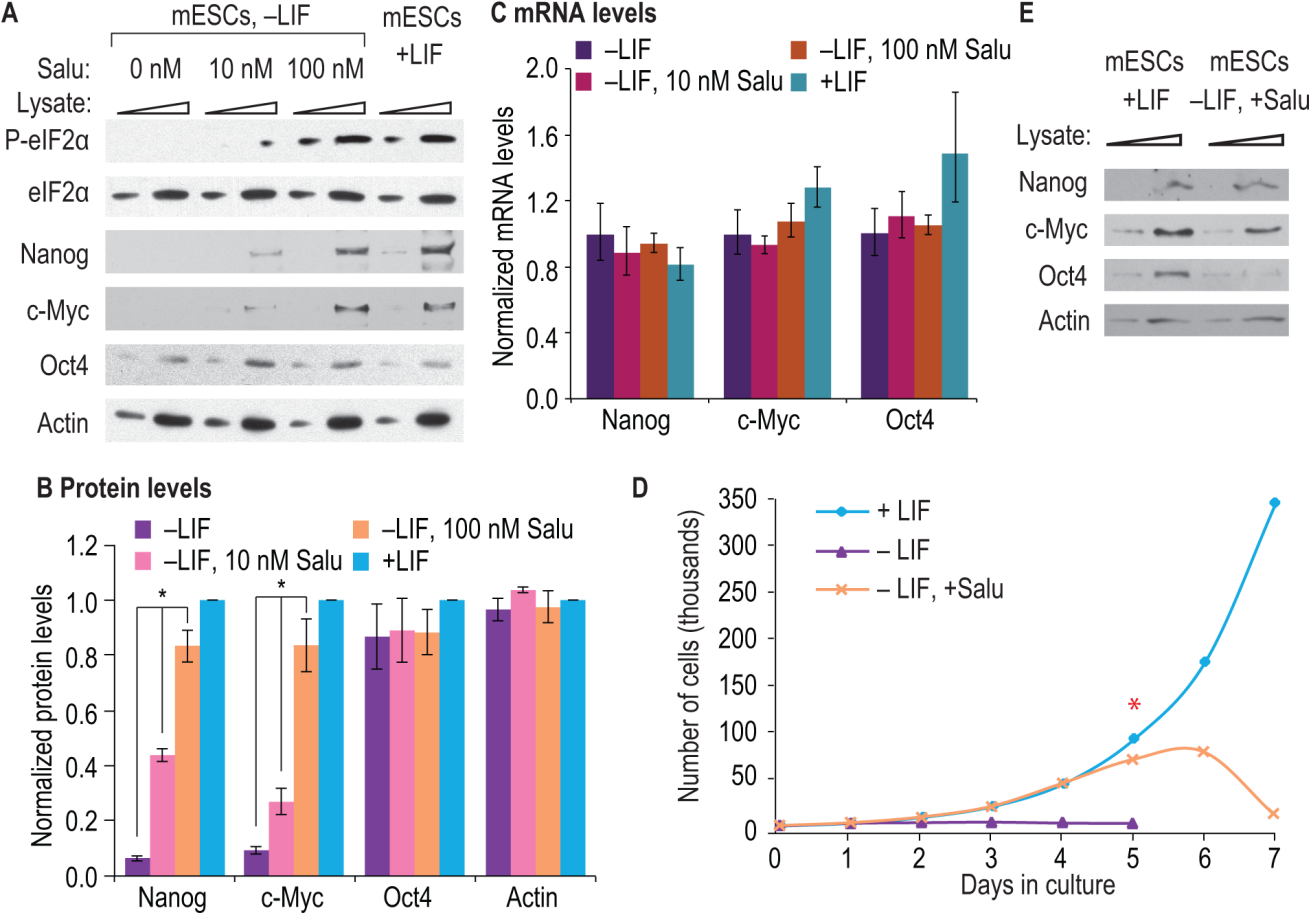
Salubrinal, a CReP inhibitor, prolongs Nanog and c-Myc expression. **(**A) mESCs were cultured as in Fig 3 with addition of salubrinal at 0 nM, 10 nM and 100 nM to mESCs cultured without LIF. Increasing amounts of cell lysate (indicated by triangles) were separated by SDS-PAGE and western blotted for indicated proteins. Treatment with salubrinal (Salu) increased the abundance of P-eIF2α, but had no effect on total eIF2α; salubrinal treatment also increased Nanog and c-Myc protein levels, but not Oct4 or Actin. (B) Three biological replicates were prepared as in (A) and quantitated. Data were then normalized to values from mESCs grown with LIF. Error bars represent s.d. (*, p < 0.01). (C) RNA was prepared from mouse ESCs cultured as in (A) and RT-qPCR used to assay abundance of indicated RNAs. All data were normalized to a control probe set (eEF1A mRNA) and mESCs grown with LIF. Nanog, c-Myc and Oct4 mRNA levels were unaltered in mESCs grown in various combinations of LIF and salubrinal (Salu). Error bars represent s.d. (*, p < 0.01 compared to mESCs–LIF samples). (D) Mouse ESCs were cultured with LIF (+LIF), without LIF (–LIF) or without LIF but with salubrinal (–LIF, +Salu) over several days. Cell numbers were counted on each day of culture. Mouse ESCs grown with LIF continued to divide (aqua line), whereas mESCs cultured without LIF (–LIF) rapidly stop dividing (purple line). Mouse ESCs cultured without LIF, but with salubrinal (–LIF, +Salu) (orange line) divided normally for several days but then senesced with the appearance of cell corpses suggesting apoptosis. (E) Mouse ESCs were cultured with LIF or without LIF, but with 100 nM salubrinal, for 5 days. Increasing amounts of cell lysate (indicated by triangles) was separated by 4–12% gradient SDS-PAGE and western blotted for indicated proteins. Nanog, c-Myc and Oct4 were all present in mESCs cultured with LIF. In mESCs cultured without LIF, but with added salubrinal, Nanog and c-Myc proteins were expressed at similar levels as in mESCs cultured in LIF. Oct4 levels were higher in mESCs cultured with LIF compared to mESCs cultured without LIF but with salubrinal. Actin was probed as a loading control. Therefore, salubrinal is sufficient to maintain Nanog and c-Myc protein expression, but cannot preserve Oct4 expression.

Mouse and human ESCs were collected using trypsin-EDTA to detach the cells from tissue culture plates. Cells were then washed twice with PBS and resuspended in a minimal volume of SDS-PAGE loading dye. Dilutions (for Figs 1A, 1E, 2A and 3A: 1 μL, 3 μL and 9 μL; Figs 1C, 2C and 4A: 2 μL and 6 μL) were then loaded onto 4–12% SDS-PAGE gradient gels and electrophoresed. Samples were then transferred to nitrocellulose and blotted with the indicated antibodies. For phosphospecific antibodies, membrane blocking and primary antibody incubations were performed in 5% (w/v) BSA in PBS with 0.5% Tween-20 (PBSTw). Secondary antibodies were probed in 5% (w/v) nonfat dry milk in PBSTw. Quantitation was performed using an ImageQuant LAS4000 (GE Healthcare, Little Chalfont, UK) and ImageQuant software. For quality control, multiple dilutions were used to ensure that blots were in the linear range, and data were normalized as indicated in the relevant figures. In all cases, reported standard deviations were calculated from biological replicates (minimum three separate trials).

### Immunoprecipitation

Mouse ESCs were collected using trypsin-EDTA, washed twice with PBS and resuspended in 300 μL PKR immunoprecipitation buffer [10 mM Tris-HC1, pH 7.7, 50 mM KCl, 2 mM MgCl_2_, protease inhibitors, 1 mM PMSF, 1 mM DTT [27]]. Lysate was precleared at 12,000 rpm for 5 min at 4°C and added to anti-PKR antibody immobilized on protein A-agarose beads (Pierce Biotechnology, Rockford, IL). Beads alone were used as a control. Samples were incubated at 4°C for 4 h and washed 6 times with ice-cold 300 μL PKR immunoprecipitation buffer. Samples were then separated by 4–12% SDS-PAGE and western blotted with the indicated antibodies.

### Immunofluorescence

Mouse ESCs were cultured as described but were grown in chamber slides. Prior to fixation, cells were washed in PBS twice and were fixed with 3% paraformaldehyde dissolved in 100 mM K_2_HPO_4_ (pH 7.2) for 30 min at room temperature. Samples were then washed twice with ice-cold PBSTw, and ice-cold methanol was added. Samples were then stored at -20°C for at least 15 min. Samples were washed 3 times with ice-cold PBSTw and blocked for 30 min at room temperature in PBSTw + 0.5% BSA. Samples were then incubated with primary antibody (as indicated in figures) at a 1:100 dilution in PBSTw + 0.5% BSA for three h at room temperature. Samples were washed 3 times with ice-cold PBSTw + 0.5% BSA for 15 min each at room temperature. Samples were then incubated with DAPI and the appropriate secondary antibody (at 1:500 dilution) conjugated to a fluorophore for two h at room temperature. Samples were washed 3 times with ice-cold PBSTw + 0.5% BSA for 15 min each at room temperature. Samples were then imaged on a Leica SP8 confocal microscope. M phase cells were quantitated by counting cells with clear metaphase plates or chromosomes in anaphase. Three biological replicates were quantitated separately.

### RT-qPCR

Mouse ESCs were cultured as described, and total RNA was extracted with Trizol Reagent according to manufacturer’s instructions (Life Technologies, Carlsbad, CA). cDNA synthesis was performed with oligo-dT primers and 5 μg total RNA using Superscript III reverse transcriptase again according to manufacturer’s instruction (Life Technologies, Carlsbad, CA). qPCR was then performed on an Applied Biosystems 7500 Fast Real-Time PCR System (Life Technologies, Carlsbad, CA). The Taqman assay probe sets used were mouse Nanog (Mm016117761_g1), c-Myc (Mm00487804_m1), Oct4 (Mm03053917_g1) and human eEF1A (also amplifies mouse; Hs00265885_g1). In each case, data were normalized to the internal control, eEF1A. Technical replicates were performed in each case, but reported standard deviations are for independent, biological replicates (done three times).

## Results

### Mouse and human ESCs contain phosphorylated eIF2α and eIF4G

Translation initiation is regulated by phosphorylation of multiple initiation factors, which were analyzed earlier [6]. However, in these previous experiments, mESCs were not cultured in defined medium, making it impossible to explore the role of specific growth factors. Mouse ESCs can be maintained in defined medium supplemented with LIF and BMP4 [28], with growth factor removal promoting neural differentiation [28]. We therefore asked whether key translation initiation factors were differentially phosphorylated in ESCs grown in the presence or absence of LIF. Note that for routine mouse ESC culture, LIF was always included. We selected a time point of 24 hours after LIF removal because LIF-cultured mESCs maintain the pluripotency marker Rex1 as well as colony-forming capacity one day after LIF is withdrawn [29]. Protein was extracted, and phosphospecific antibodies were used to detect phosphorylated eIF2α, eIF4G and eIF4E, three translation factors regulated by phosphorylation [30–32]. P-eIF2α and P-eIF4G were more abundant in mouse ESCs grown with LIF (Fig 1A, +LIF) than in those cultured for one day without LIF (Fig 1A,–LIF); by contrast, P-eIF4E was present at a similar level with or without LIF (Fig 1A). Yet levels of total eIF2α, eIF4G, eIF4E, and Actin (loading control) were the same with or without LIF (Fig 1A). The result that LIF maintains phosphorylated eIF2α and eIF4G in mouse ESCs was reproducible (Fig 1B).

We also asked how P-eIF2α and P-eIF4G respond to changes in LIF concentration. To this end, we cultured mESCs with increasing LIF concentrations for 24 hours (note that routine mESC culture uses 10 ng/mL LIF) and then assayed P-eIF2α and P-eIF4G as well as total eIF2α and eIF4G proteins. As LIF increased, P-eIF2α and P-eIF4G increased (Fig 1C, from left to right), but total eIF2α, eIF4G, and Actin did not change. Quantitation of multiple experimental trials confirmed this result (Fig 1D). We also note that high P-eIF4G levels were maintained at a lower LIF concentration than was true for P-eIF2α, suggesting that LIF may promote phosphorylation of these two translation factors in distinct ways.

Human and mouse ESCs differ in growth factor requirements (reviewed in [33]). We therefore asked if human ESCs also contain P-eIF2α and P-eIF4G when cultured with TGF-β and FGF2, which maintain their pluripotency [25], or when cultured under conditions that drive their differentiation to mesendoderm [26]. ESCs treated with TGF-β and FGF2 (Pluri.) possessed considerably more P-eIF2α and P-eIF4G than those induced to differentiate (Diff.) (Fig 1E), a result confirmed in repeated trials (Fig 1F). We conclude that eIF2α and eIF4G are phosphorylated in both human and mouse ESCs and that their phosphorylation decreases upon differentiation. We focused on eIF2α for the remainder of our studies because Nanog and c-Myc mRNAs both possess uORFs and therefore may be subject to eIF2α regulation.

### LIF inhibits eIF2α dephosphorylation

Abundant P-eIF2α depends on LIF (Fig 1). We postulated that LIF might promote high P-eIF2α, either by activating an eIF2α kinase or by inhibiting a P-eIF2α phosphatase. Among four related eIF2α kinases in mammals–GCN2, PKR, PERK and HRI [18, 20, 30, 34], we focused on PKR as the likely kinase. The other kinases rely on special conditions for activity: GCN2 and PERK respond to cellular stresses such as amino acid starvation and oxidative stress [18, 19], and HRI is activated by heme depletion [35]. Similarly, among the two P-eIF2α phosphatases, we focused on the PP1-CReP-Nck1/2 heterotrimeric phosphatase, because it is present constitutively rather than inductively [22, 36]. To ask if LIF affects either the PKR kinase or the PP1-CReP-Nck1/2 phosphatase, we cultured mESCs with or without LIF for one day, harvested cells, and separated lysate by SDS-PAGE. We then western blotted for PKR, which is phosphorylated upon activation [37], and for the CReP and Nck1/2 subunits of the phosphatase. Similar levels of total and phosphorylated PKR (Thr446) were present with or without LIF (Fig 2A), suggesting that PKR phosphorylation is independent of LIF. By contrast, CReP abundance was low in the presence of LIF, but high after LIF withdrawal (Fig 2A). LIF had no effect on either Nck1/2 or Actin, which was probed as a loading control (Fig 2A). The low CReP levels in mESCs treated with LIF were reproducible (Fig 2B).

We also asked how CReP abundance responds to changes in LIF concentration. Mouse ESCs were cultured with increasing LIF concentrations for 24 h (as above, note that routine mESC culture uses 10 ng/mL LIF) and CReP protein was monitored by western blotting. Increased LIF correlated with decreased CReP protein (Fig 2C). Nck1/2 and Actin were again unchanged (Fig 2C) and served as loading controls. Quantitation of multiple blots confirmed this result (Fig 2D). Importantly, the decrease in CReP abundance occurred at the same LIF concentration that P-eIF2α increased (compare Figs 1D and 2D). CReP expression is regulated at the mRNA and protein levels [23]; which step(s) is regulated by LIF is a future avenue of research. Since CReP delivers the phosphatase activity to eIF2α [22], the simplest interpretation is that LIF maintains abundant P-eIF2α in mESCs by lowering abundance of the CReP mRNA or protein.

### BMP4 activates eIF2α phosphorylation

Mouse ESC pluripotency is maintained by BMP4 in addition to LIF [28, 38]. We next postulated that BMP4 might activate an eIF2α kinase to complement LIF. To test this idea, we cultured mESCs for one day under three conditions: LIF and BMP4, BMP4 but no LIF, or LIF but no BMP4. We again detected P-PKR in either the presence or absence of LIF (Fig 2E). However, without BMP4, PKR phosphorylation was lost while total PKR levels remained constant (Fig 2E). Importantly, both LIF and BMP4 were required for the normally high levels of P-eIF2α (Fig 2E). Activated P-PKR can also be monitored by its substrate binding [39], so we next used immunoprecipitation (IP) to query PKR activity. Specifically, we immunoprecipitated PKR from mESCs cultured as above and then assayed the IPs for eIF2α. When mESCs were cultured with BMP4 (with or without LIF), PKR associated with eIF2α (Fig 2F); however, when mESCs were cultured without BMP4 (plus LIF), PKR failed to associate with eIF2α (Fig 2F). In all cases, the total quantity of immunoprecipitated PKR remained constant (Fig 2F). Therefore, BMP4 signaling leads to PKR phosphorylation and drives PKR to associate with its eIF2α substrate, although additional eIF2α kinases may also be involved. It is important to note that BMP4 is necessary but not sufficient to increase P-eIF2α levels since LIF is required to destabilize the eIF2α phosphatase, CReP. Together, our results suggest that the LIF and BMP4 growth factors use distinct but complementary mechanisms to promote eIF2α phosphorylation: LIF decreases CReP, a part of the P-eIF2α phosphatase complex, while BMP4 activates PKR, the eIF2α kinase.

### P-eIF2α promotes expression of pluripotency factors Nanog and c-Myc

P-eIF2α can promote CDS expression from mRNAs harboring uORFs (see Introduction). Nanog and c-Myc mRNAs are potential targets of eIF2α regulation since both mRNAs contain translated uORFs [40]. Based on our finding that pluripotent ESCs possess abundant P-eIF2α, whereas ESCs triggered to differentiate do not (Fig 1), we asked whether P-eIF2α affects Nanog or c-Myc expression. We also assayed Oct4 expression since Oct4 protein is a pluripotency factor, but Oct4 mRNA does not contain a translated uORF [10]. As a baseline, we first monitored Nanog and c-Myc protein and mRNA levels in mESCs. As described above, mESCs were cultured in the presence or absence of LIF for one day, and western blots were performed for Nanog, c-Myc, and Oct4 [41]. As expected, Nanog and c-Myc proteins were more abundant in mESCs grown in LIF than in mESCs grown without LIF, as previously described [11, 12] (Fig 3A). By contrast, Oct4 protein levels were comparable, but modestly decreased, in mESCs grown with or without LIF (Fig 3A), consistent with the work of others [12]. Quantitation confirmed this result (Fig 3B). We next monitored Nanog, c-Myc and Oct4 mRNA levels in mESCs treated as above. Using RT-qPCR (normalized to a control eEF1α mRNA), Nanog, c-Myc and Oct4 mRNA abundances were constant, with or without LIF (Fig 3C). The decline in Nanog and c-Myc protein abundance without a drop in mRNA levels suggests that Nanog and c-Myc expression relies either on control of translation or protein stability. We also asked if Nanog and c-Myc proteins were expressed throughout the cell cycle since LIF withdrawal initially arrests the cell cycle [42]. Immunofluorescence detected Nanog, c-Myc and Oct4 proteins within all cells in the colony (Fig 3D). We attempted to use ribosomal profiling to see a shift from uORF to CDS translation in Nanog and c-Myc mRNAs when cultured in LIF, but a lack of uORF sensitivity in this assay precluded this approach (data not shown). We conclude that LIF increases the abundance of Nanog and c-Myc proteins without changing abundance of their mRNAs.

To test whether LIF affects Nanog and c-Myc protein expression via its control of P-eIF2α, we turned to a chemical agent that induces eIF2α phosphorylation. The drug salubrinal inhibits CReP with high specificity and also increases P-eIF2α levels [24]. We cultured mESCs with or without LIF for 24 h, but this time added salubrinal at lower (10 nM) and higher (100 nM) concentrations to the mESCs grown without LIF. Salubrinal should substitute for LIF if the LIF-dependent increase in P-eIF2α (Fig 1) results from a LIF-dependent decrease in the CReP eIF2α phosphatase (Fig 2A–2D). Indeed, salubrinal could maintain P-eIF2α at a level equivalent to that induced by LIF (Fig 4A, bottom panels), and salubrinal promoted Nanog and c-Myc protein expression (Fig 4A, top panels). Yet no significant change was found for Oct4, total eIF2α, or the Actin control (Fig 4B). Quantitation confirmed these results (Fig 4C). Although salubrinal treatment increased Nanog and c-Myc protein levels (Fig 4C), the drug did not perturb mRNA levels (Fig 4C). Salubrinal likely mediates its effects via increased P-eIF2α, but one caveat, as with any chemical treatment, is the possibility of off-target effects. Minimally, salubrinal can substitute for LIF to maintain the abundance of Nanog and c-Myc proteins in mESCs.

We next explored whether salubrinal treatment might prolong mESC pluripotency. Normally LIF withdrawal rapidly arrests cell divisions in mESCs [42], so we first asked if mESCs cultured with salubrinal continued to divide upon LIF withdrawal. Indeed, salubrinal prolonged cell divisions for about five days in mESCs grown without LIF (Fig 4D) and also maintained Nanog and c-Myc protein for a similar period (Fig 4E). However, as might be expected, salubrinal did not maintain Oct4 protein expression during that interval (Fig 4E), and cell corpses became common after seven days. Oct4 promotes Nanog transcription [43], and Oct4 loss in other systems results in ESC differentiation [44, 45]. Under our culture conditions, Oct4 loss is a likely culprit for ESC senescence. We conclude that salubrinal prolongs mESC cell divisions and maintains Nanog and c-Myc protein expression in the absence of LIF, but that salubrinal does not substitute for LIF to maintain long term pluripotency.

## Discussion

**Fig 5:**
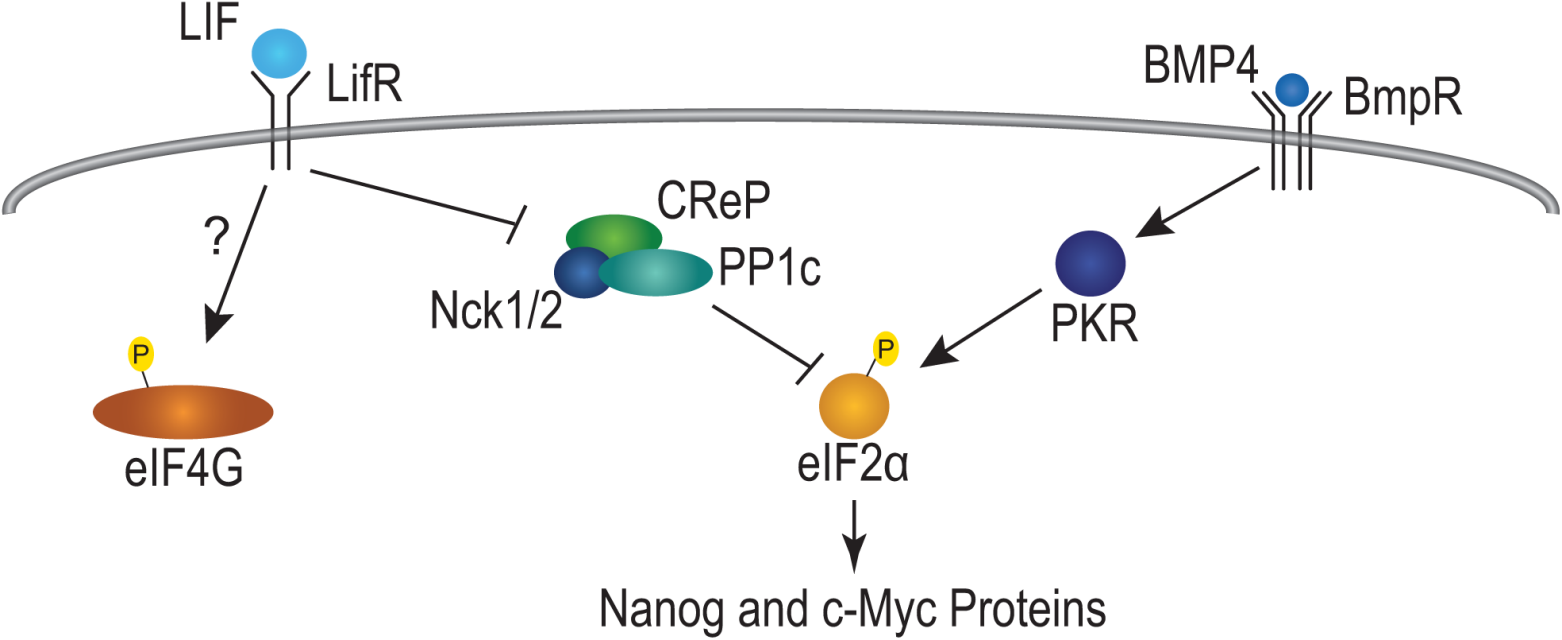
LIF and BMP4 regulate eIF2α phosphorylation and translation initiation. A model for how LIF and BMP4 regulate translation initiation. LIF and BMP4 bind receptors on the cell surface leading to decreased CReP and increased PKR activity respectively. LIF signals to phosphorylate eIF4G via an unknown pathway. CReP loss and PKR activation increase P-eIF2α levels.

The major finding of this work is that growth factors regulate translation initiation in ESCs, as summarized in Fig 5. Briefly, LIF and BMP4 maintain phosphorylated eIF2α in murine ESCs, and separate growth factors maintain P-eIF2α in human ESCs. P-eIF2α correlates with high Nanog and c-Myc expression; intriguingly, the drug salubrinal can also drive eIF2α phosphorylation and bypass the need for LIF to maintain Nanog and c-Myc protein expression. However, salubrinal does not substitute for LIF indefinitely since Oct4 levels eventually decrease in cells cultured without LIF. An open question is what role phosphorylated eIF4G may play in stem cell maintenance. One possibility is that P-eIF4G may be mediating its affects via enhanced interaction with eIF3. The mammalian target of rapamycin complex 1 (mTORC1) promotes eIF4G and eIF3 association [46], and P-eIF4G levels are sensitive to rapamycin [47]. Together, these findings demonstrate that growth factors promoting ESC pluripotency affect eIF2α phosphorylation and the translational network in ESCs.

### Connections between ESC growth factors and phosphorylated eIF2α

LIF and BMP4 can maintain mESC pluripotency [28], and our results now link those growth factors to maintaining abundant P-eIF2α by distinct mechanisms. LIF reduces expression of the eIF2α phosphatase, CReP, and BMP4 activates phosphorylation of the eIF2α kinase, PKR.

But open questions remain. For one, how does LIF reduce CReP expression? In mESCs, LIF activates Jak1 via the LIF receptor [48], which allows Jak1 to phosphorylate Stat3, driving it into the nucleus [45]. So, CReP may be a substrate for the Jak1 kinase. Another question is whether there might be an additional connection between LIF and PKR. This idea is based on the finding in another cell type that Stat3 regulates PKR. Specifically, in U2OS osteosarcoma cells, a high level of cytoplasmic Stat3 inhibits PKR [49]. If the same mechanism were true in ESCs, then LIF uses a two-pronged attack to increase eIF2α phosphorylation, both by activating PKR as Stat3 is sent to the nucleus in a phosphorylation-independent pathway (based on the work of Shen et al. [49]) and by destabilizing CReP, the eIF2α phosphatase (based on our work, see Fig 2). We also find that BMP4 induces PKR phosphorylation, but the mechanism is unknown. Work from others provides ideas. PKR can be complexed with TAK1, TAB1 and TAB2 [50], proteins that function in BMP signaling [51, 52]. In addition, BMP2 activates PKR expression and phosphorylation in human MCF-7 cells [53]. So, does PKR association with TAK1, TAB1 and TAB2 mediate its BMP-dependent activation? These questions are avenues of future research.

Human ESCs are typically maintained with distinct growth factors from mouse ESCs. Routine hESC culture uses FGF2 and TGF-β [25], and we report here that hESCs grown in this routine way contain abundant P-eIF2α (see Fig 1). Intriguingly, FGF2 and TGF-β have been connected to P-eIF2α in other systems. In smooth muscle, FGF2 promotes eIF2α phosphorylation via the phosphatidylinositol 3-kinase pathway [54], a pathway known to function in hESCs to promote pluripotency [55]. In eosinophils, TGF-β activates PKR phosphorylation [56]. Neither with FGF2 nor TGF-β has the mechanism connecting growth factor to eIF2α phosphorylation been determined. On a separate note, BMP4 promotes hESC differentiation, rather than maintenance [57]. A separate possibility is that PKR is not the relevant eIF2α kinase in human ESCs (as compared to our findings with mouse ESCs). It will be of interest to flesh out the full regulatory mechanism that explains how FGF2 and TGF-β converge on eIF2α phosphorylation in hESCs. A further question is whether the mechanism that increases P-eIF2α in human ESCs is shared in mouse ESCs.

### Phosphorylated eIF2α may normally rely on hypoxia rather than growth factors

Prior to implantation and oxygenation from the maternal blood supply, embryos live in a hypoxic environment [58]. In fact, hypoxic conditions drive a higher percentage of human embryos to reach the blastocyst stage when cultured *in vitro* [59]. Most relevant to this work, hypoxia induces eIF2α phosphorylation [19], along with many other cellular responses. Given the profound regulatory role of phosphorylated eIF2α (reviewed in [60]), we suggest that a critical function of ESC growth factors is to preserve eIF2α phosphorylation in ESCs cultured at atmospheric oxygen concentrations. It will be of great interest to see whether eIF2α phosphorylation can be manipulated by chemical agents such as salubrinal to improve ESC derivation, induced pluripotency or ESC maintenance in the future.
